# Effect of SGLT-2 inhibitor, empagliflozin, on blood pressure reduction in Chinese elderly hypertension patients with type 2 diabetes and its possible mechanisms

**DOI:** 10.1038/s41598-022-07395-x

**Published:** 2022-03-03

**Authors:** Lan Cheng, Qianyu Fu, Longhua Zhou, Yuqin Fan, Fenfen Liu, Yuanyuan Fan, Xin Zhang, Weiqing Lin, Xiaohe Wu

**Affiliations:** Jiangxi Provincial People’s Hospital, Nanchang, 330006 Jiangxi China

**Keywords:** Type 2 diabetes, Hypertension

## Abstract

The current study evaluated the effect of SGLT-2 inhibitor, empagliflozin, on blood pressure reduction in Chinese elderly hypertension patients with type 2 diabetes and investigated its possible mechanisms. 124 patients were randomized to receive 25 mg empagliflozin QD, or placebo double blind for 12 weeks. Patients underwent 24-h ABPM. Endothelial function and arterial stiffness were also measured prior to randomization and at week 12. At week 12, adjusted mean difference versus placebo in change from baseline in mean 24-h SBP was − 8.14 mmHg (95% CI − 10.32, − 3.96, *P* = 0.005). At week 12, adjusted mean difference versus placebo in change from baseline in mean 24-h DBP was − 5.27 mmHg (95% CI − 8.19, − 1.35, *P* < 0.001). Changes in office BP were consistent with ABPM. Empagliflozin was well tolerated. Empagliflozin was associated with significant and clinically meaningful reductions in BP versus placebo in Chinese elderly patients with type 2 diabetes and hypertension. The underlying mechanisms possiblely at least in part were the improvements of endothelial function and arterial stiffness associated with empagliflozin.

Registration number: ChiCTR2100054678, Registration date: December 23, 2021.

## Introduction

Type 2 diabetes and hypertension are the most common non-communicable diseases in elderly people^1^ and they also are the major causes of death in population over 60 years-old^2^. Prevalence of hypertension in patients with diabetes is 2 times that of non-diabetic people. Meanwhile, prevalence of simultaneous diabetes and hypertension in elderly people is 1.5 times that of young people^3^. Both hypertension and diabetes are cardiovascular disease risk factors. The cardiovascular disease risk is 3 times in patients with type 2 diabetes compared with non-diabetic subjects^4^. In addition, 1.7 fold and 1.5 fold increased risk for all-cause mortality and cardiovascular events, respectively, are shown in diabetic individuals with hypertension compared with those with normal blood pressure^5^. Tight blood pressure control could decrease risk of macrovascular and microvascular complications in type 2 diabetes by 20–40 percent^3^*.* Studies have indicated that the adverse effects of hypertension and diabetes may be addictive^6,7^, making the treatments targeted both glycemic and blood pressure control are crucial options for physicians.

Traditional antihypertension drugs, such as diuretics and β-adrenoceptor blockers, the effects of which on glycaemic control are negative. Thiazide diuretics inhibits insulin function by acting on ionic channel in cell membranes, decreasing intracellular magnesium and potassium contents, reducing glucose transporter protein expression^8^. Beta-blockers increase the incidence of diabetes by 28 percent, impairing blood supply and insulin sensitivity in tissue^9^. Although ACEI/ARB could improve insulin resistance, high prevalence of persistent cough and angioneurotic edema induced by them urges us to find a new efficient and safe antihypertension drug in patients with diabetes.

Some hypoglycemic drugs, such as GLP-1 receptor agonists, DPP-4 inhibitors and thiazolidinediones, have a slight antihypertensive action. SGLT-2 inhibitors is a new antidiabetic drug class which lower blood glucose by promoting the renal excretion of glucose. Some studies have indicated that they also have hypotensive effect^10^. Multiple mechanisms involve BP reduction by SGLT-2 inhibitors, including osmotic diuresis, mild natriuresis, body weight loss, local inhibition of the RAAS^11^, and nitric oxide release^12^. Whether there are other relevant mechanisms related to the BP-lowering effect of SGLT-2 inhibitors is still elusive.

The current study evaluated the effect of SGLT-2 inhibitor, empagliflozin, on blood pressure reduction in elderly hypertension patients with type 2 diabetes and investigated its possible mechanisms.

## Study population

Patients with type 2 diabetes and hypertension (mean seated office SBP 140–179 mmHg or DBP 90–109 mmHg) aged 65–80 years with a BMI ≤ 30 kg/m^2^ and HbA1c 7.0–10.0% at screening were eligible for inclusion. Previous antidiabetes and antihypertension therapies had remained unchanged after enrollment. Key exclusion criteria included uncontrolled hyperglycemia (fast plasma glucose ≥ 13.3 mmol/L), mean seated SBP ≥ 180 mmHg and/or mean seated DBP ≥ 110 mmHg, known/suspected secondary hypertension, malignant hypertension, history/evidence of hypertensive retinopathy or hypertensive encephalopathy, renal impairment (estimated glomerular filtration rate [eGFR] ≤ 60 mL/min/1.73 m^2^,using the MDRD equation), or indication of liver disease (serumalanine aminotransferase, aspartate aminotransferase, or alkaline phosphatase more than three times the upper limit of normal), history of acute coronary syndrome, stroke, or transient ischemic attack.

### Study design and treatment

Patients were randomized (1:1) to receive 25 mg empagliflozin QD, or placebo double blind for 12 weeks. Randomization was undertaken using a computer-generated numbers and sealed envelopes were used for allocation concealment. Patients continued their antihypertensive and antidiabetes background therapy throughout the trial at an unchanged dose and regimen. Office BP was measured using Omron M digital BP monitor by three physicians. Three measurements after resting in a quiet and temperature-controlled room were performed and average was recorded. ABPM was performed by ABPM lab in the hospital using Spacelabs Ultralite 90217 devices in accordance with manufacturer recommendations and in agreement with current ESC/ESH Guidelines. The ABPM devices were programmed to measure BP and pulse every 30 min. Patients underwent 24-h ABPM ≤ 7 days prior to randomization and at week 12. At both times, patients’ daytime and nighttime activities during the 24 h were to be similar. Blood sample was collected and sent to assay in the clinical lab of the hospital for fasting plasma glucose, serum insulin and HbA1c. HOMA-IR score was calculated using the HOMA-IR formula (HOMA-IR = fasting insulin (mIU/l) × fasting glucose (mmol/l)/22.5. Glomerular filtration rate was estimated using the Modification of Diet in Renal Disease Study equation.

Patients underwent 24-h ABPM, endothelial function assessment [brachial artery flow-mediated dilation (FMD)], arterial stiffness assessment [carotid artery pulse wave velocity (cPWV)] prior to randomization and at week 12. Endothelial function was assessed in accordance with current guidelines through brachial artery FMD. Patients laid supine with their right arm extended and fixed on the examination table with rubber foam. Blood pressure was recorded using the other arm. An additional blood pressure cuff was used on the right forearm. The brachial artery was recorded by ultrasound and its diameter was obtained by using the machine’s continuous tracking software. Baseline diameter was obtained for 1 min, and after that, the blood pressure cuff was inflated to 50 mmHg above the systolic blood pressure for 4 min, resulting in the occlusion of the forearm arteries. After that time, the blood pressure cuff was rapidly deflated, inducing reactive hyperaemia. Te brachial artery diameter was then continuously recorded for another 3 min. At the end of examination, the ultrasound machine automatically generated the values of baseline and maximal brachial artery diameter and FMD. cPWV measurement was performed on the right common carotid artery. Patients laid in a supine position, their head additionally elevated by 45° and tilted to the side by 30°. Using the device’s software, stiffness was measured through analysis of the pulse waves obtained. The tracker pair was fixed at the anterior and posterior wall of the common carotid artery. The machine obtained the pressure wave forms from the changing arterial diameters that were calibrated based on systolic and diastolic blood pressure. Then cPWV was calculated automatically as a mean of 12 beats.

The protocol was approved by the ethics committee of Jiangxi Provincial People’s Hospital. The study was conducted in accordance with the principles of the Declaration of Helsinki. All patients provided written informed consent before enrollment into the study.

### Statistical methods

A sample size of 60 patients per treatment group would provide a power of 90% to detect a treatment difference of 8 mmHg (SD 13) in 24-h SBP at a significance level of 5% (two-sided), and a 5% dropout rate. This sample size would have 97% power to detect a 5 mmHg difference in mean 24-h DBP assuming an SD of 7 mmHg.

Baseline characteristics are expressed as the number of observations and percentage for categorical variables or the median ± SEM for continuous variables. Differences between groups were assessed with the independent-samples *t* test for continuous variables and the χ^2^ test for categorical variables. For the clinical endpoints (change in various indices from baseline to week 12), the analysis of covariance was used; the results were expressed as adjusted mean (standard error). Analysis of covariance included the baseline value of each analyzed variable as covariates. SPSS22.0 statistical software was used. *P* < 0.05 was statistically significant.

## Results

Between September 2020 and March 2021, 124 elderly patients with type 2 diabetes and hypertension were randomized and received study medication. Patient baseline characteristics are shown in Table 1. The two groups of patients had similar baseline characteristics. No significant differences in systolic and diastolic blood pressure (office measurement or mean over 24 h) were found between the groups. Patients with isolated systolic hypertension accounted for the whole study population by 27.4% (17 cases) and 22.5% (14 cases) in placebo group and empagliflozin group, respectively.

**Table 1:** Patient baseline characteristics.

	Placebo n = 62	Empagliflozin n = 62	*P* value
Male sex, n (%)	37 (59.7)	40 (64.5)	0.579
Patients with isolated systolic hypertension, n (%)	17 (27.4)	14 (22.5)	0.534
Age, years	71.7 ± 4.3	71.2 ± 4.0	0.560
BMI, kg/m^2^	25.8 ± 2.3	25.5 ± 2.3	0.565
eGFR, mL/min/1.73 m^2^	81.5 ± 8.6	83.0 ± 7.0	0.285
HbA1c, %	7.7 ± 1.1	7.7 ± 0.5	0.887
FPG, mmol/L	8.6 ± 1.0	8.4 ± 0.8	0.252
**Years since diagnosis of type 2 diabetes, n (%)**			
≤ 1	7 (11.3)	8 (12.9)	0.064
> 1 to 5	19 (30.6)	11 (17.7)	
> 5 to 10	22 (35.5)	16 (25.8)	
> 10	14 (22.6)	27 (43.5)	
SBP (office measurement), mmHg	159.5 ± 8.0	157.1 ± 10.1	0.151
SBP (mean over 24 h), mmHg	150.8 ± 37.2	153.0 ± 36.8	0.384
DBP (office measurement), mmHg	97.7 ± 4.7	98.5 ± 2.0	0.278
DBP (mean over 24 h), mmHg	95.4 ± 22.3	96.2 ± 23.4	0.171
Pulse rate (mean 24 h), bpm	77.8 ± 5.2	77.3 ± 8.2	0.705
Serum insulin (uU/mL)	14.2 ± 2.9	14.4 ± 3.7	0.704
HOMA-IR	5.5 ± 1.4	5.4 ± 1.5	0.828
FMD (%)	7.1 ± 1.0	7.0 ± 1.0	0.289
cPWV(m/s)	6.7 ± 1.4	6.4 ± 0.8	0.085
**Antihypertensive drugs**			
Diuretic drugs	10 (16.1)	12 (19.4)	0.638
Calcium channel blockers	25 (40.3)	29 (46.8)	0.469
ACEI/ARB	43 (69.4)	45 (72.6)	0.692
β-blockers	27 (43.5)	32 (51.6)	0.369
**Anti-diabetic drugs**			
Insulin	7 (11.3)	11 (17.7)	0.308
Sulfonylurea	14 (22.6)	19 (30.6)	0.310
DPP4 inhibitor	27 (43.5)	31 (50.0)	0.472
Metformin	47 (75.8)	43 (69.4)	0.421
**Lipid-lowering agents**			
Statins	45 (72.6)	50 (80.6)	0.289
Fibrates	13 (21.0)	19 (30.6)	0.218
Patients with BP ≥ 130/80 mmHg (ABPM) at baseline, Mean 24-h SBP	186.1 ± 21.4	189.5 ± 21.6	0.895
Patients with BP ≥ 130/80 mmHg (ABPM) at baseline, mean 24-h DBP	114.7 ± 23.9	116.1 ± 24.1	0.571
Patients with BP < 130/80 mmHg (ABPM) at baseline, mean 24-h SBP	123.5 ± 5.7	121.0 ± 8.1	0.366
Patients with BP < 130/80 mmHg (ABPM) at baseline, mean 24-h DBP	71.7 ± 6.9	72.5 ± 5.2	0.277

**Figure 1:**
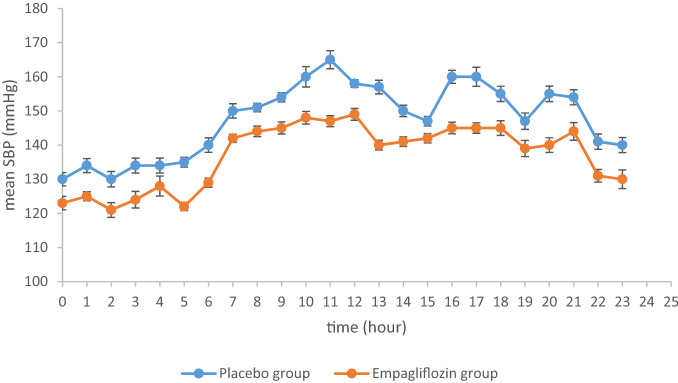
Hourly mean SBP (APBM) at week 12.

**Figure 2:**
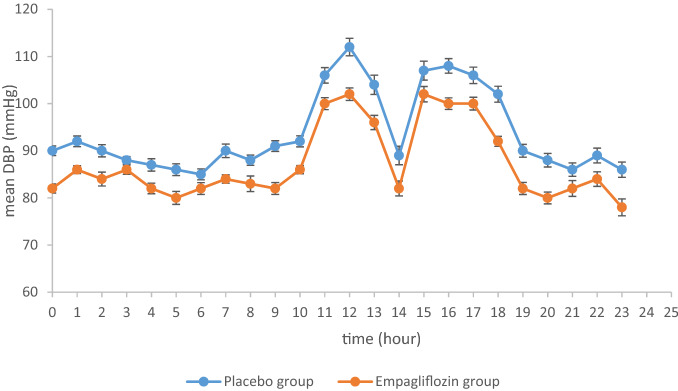
Hourly mean DBP (APBM) at week 12.

Hourly mean SBP and DBP at week 12 are shown in Figs. 1 and 2.

At week 12, adjusted mean difference versus placebo in change from baseline in mean 24-h SBP was -8.14 mmHg (95% CI − 10.32, − 3.96, *P* = 0.005). At week 12, adjusted mean difference versus placebo in change from baseline in mean 24-h DBP was − 5.27 mmHg (95% CI − 8.19, − 1.35, *P* < 0.001). Similarly, at week 12, adjusted mean difference versus placebo in change from baseline in office SBP was − 6.27 mmHg (95% CI − 9.37, − 1.97, *P* < 0.001). Adjusted mean difference versus placebo in change from baseline in office DBP was − 4.47 mmHg (95% CI − 7.41, − 0.47, *P* < 0.001). Data are shown in Table 2.

**Table 2:** Change in characteristics at week 12.

	Placebo n = 62	Empagliflozin n = 62
SBP (adjusted mean over 24 h), mmHg	147.5 (0.77)	144.3 (0.77)
Change from baseline	0.41 (0.23)	− 7.73 (0.23)
Difference vs. placebo		− 8.14 (1.01)
95% CI		− 10.32, − 3.96
*P*		0.005
DBP (adjusted mean over 24 h), mmHg	94.4 (0.68)	89.1 (0.68)
Change from baseline	0.14 (0.09)	− 5.13 (0.09)
Difference vs. placebo		− 5.27 (0.07)
95% CI		− 8.19, − 1.35
*P*		< 0.001
SBP (office measurement), mmHg	155.0 (0.78)	147.9 (0.78)
Change from baseline	− 0.36 (0.12)	− 6.63 (0.13)
Difference vs. placebo		− 6.27 (0.11)
95% CI		− 9.37, − 1.97
*P*		< 0.001
DBP (office measurement), mmHg	96.4 (0.70)	90.9 (0.70)
Change from baseline	− 0.07 (0.09)	− 4.54 (0.09)
Difference vs. placebo		− 4.47 (1.01)
95% CI		− 7.41, − 0.47
*P*		< 0.001
HbA1c, %	7.6 (0.07)	7.4 (0.07)
Change from baseline	0.1 (0.09)	− 0.5 (0.09)
Difference vs. placebo		− 0.6 (0.11)
95% CI		− 0.83, − 0.42
*P*		< 0.001
Body weight, kg	76.4 (0.61)	79.6 (0.61)
Change from baseline	− 1.2 (0.99)	− 7.9 (0.99)
Difference vs. placebo		− 6.7 (0.86)
95% CI		− 8.45, − 5.03
*P*		< 0.001
HOMA-IR	4.9 (0.15)	4.5 (0.15)
Change from baseline	− 0.3 (0.09)	− 1.8 (0.09)
Difference vs. placebo		− 1.5 (0.12)
95% CI		− 1.90, − 1.06
*P*		< 0.001

At week 12, adjusted mean difference versus placebo in change from baseline in daytime mean 24-h SBP was − 10.01 mmHg (95% CI − 17.42, − 3.63, *P* < 0.001). At week 12, adjusted mean difference versus placebo in change from baseline in nighttime mean 24-h SBP was − 7.63 mmHg (95% CI − 9.03, − 1.17, *P* < 0.001). At week 12, adjusted mean difference versus placebo in change from baseline in daytime mean 24-h DBP was -6.10 mmHg (95% CI − 7.73, − 1.39, *P* < 0.001). Adjusted mean difference versus placebo in change from baseline in nighttime mean 24-h DBP was − 4.46 mmHg (95% CI − 8.80, − 0.64, *P* < 0.001). Patients with achieving target of 130/80 mmHg (ABPM) at week 12 accounted for the whole study population by 56.5% (35 cases) and 83.9% (52 cases) in placebo group and empagliflozin group, respectively. Data are shown in Table 3.

**Table 3:** Changes in daytime and nighttime BP at week 12.

	Placebo n = 62	Empagliflozin n = 62
Daytime mean SBP (ABPM), mmHg	151.5 (0.50)	142.5 (0.50)
Change from baseline	− 0.54 (0.47)	− 10.55 (0.47)
Difference vs. placebo		− 10.01 (0.70)
95% CI		− 17.42, − 3.63
*P*		< 0.001
Nighttime mean SBP (ABPM), mmHg	140.3 (0.51)	135.7 (0.51)
Change from baseline	0.11 (0.24)	− 7.52 (0.24)
Difference vs. placebo		− 7.63 (0.72)
95% CI		− 9.03, − 1.17
*P*		< 0.001
Daytime mean DBP (ABPM), mmHg	98.3 (0.60)	92.3 (0.60)
Change from baseline	− 0.19 (0.07)	− 6.29 (0.07)
Difference vs. placebo		− 6.10 (0.85)
95% CI		− 7.73, − 1.39
*P*		< 0.001
Nighttime mean DBP (ABPM), mmHg	90.7 (0.57)	82.4 (0.57)
Change from baseline	0.13 (0.06)	− 4.33 (0.06)
Difference vs. placebo		− 4.46 (0.80)
95% CI		− 8.80, − 0.64
*P*		< 0.001
**Subgroup analyses**		
**Mean 24-h SBP**		
Patients with BP ≥ 130/80 mmHg (ABPM) at baseline, n	27	30
Change from baseline	0.54 (0.29)	− 8.16 (0.29)
Difference vs. placebo		− 8.70 (0.67)
95% CI		− 10.63, − 1.67
*P*		0.002
Patients with BP < 130/80 mmHg (ABPM) at baseline, n	35	32
Change from baseline	− 0.42 (0.76)	− 8.72 (0.76)
Difference vs. placebo		− 8.30 (0.33)
95% CI		− 10.74, − 1.93
*P*		0.001
**Mean 24-h DBP**		
Patients with BP ≥ 130/80 mmHg (ABPM) at baseline, n	27	30
Change from baseline	0.39 (0.47)	− 4.87 (0.47)
Difference vs. placebo		− 5.26 (0.45)
95% CI		− 7.87, − 0.34
*P*		0.001
Patients with BP < 130/80 mmHg (ABPM) at baseline, n	35	32
Change from baseline	− 0.93 (0.97)	− 6.63 (0.97)
Difference vs. placebo		− 5.70 (0.19)
95% CI		− 8.48, − 1.09
*P*		0.004

The number of patients with AEs is summarized in Table 4. Events consistent with volume depletion were reported by one patient in empagliflozin group (hypotension and orthostatic hypotension). Comfirmed hypoglycemic AEs were reported in more patients receiving empagliflozin than placebo. The percentage of patients with events consistent with UTI was similar in the empagliflozin and placebo groups. The percentage of patients with events consistent with genital infection was higher with empagliflozin than placebo.

**Table 4:** Summary of AEs.

	Placebo n = 62	Empagliflozin n = 62
Hypoglycemia	6 (9.7)	9 (14.5)
**Events consistent with urinary tract infection**		
Male	1 (1.6)	1 (1.6)
Female	3 (4.8)	5 (8.1)
**Events consistent with genital infection**		
Male	1 (1.6)	6 (9.7)
Female	0	4 (6.5)
Events consistent with volume depletion	0	1 (1.6)

## Discussion

Hypertension is a common comorbidity in elderly patients with type 2 diabetes and increases the risk of cardiovascular complications. This study was undertaken to establish the effect of empagliflozin for 12 week on BP, metabolic control, endothelial function and arterial stiffness in elderly patients with type 2 diabetes and hypertension. We assessed both ABPM and office BP measurements because ABPM avoids the “white coat” effect seen with office BP measurements^13^. Treatment with empagliflozin for 12 weeks led to significant and clinically meaningful improvements in 24-h SBP and DBP compared with placebo, supported by reductions in office BP. The risk of cardiovascular disease doubles for each increment of 20 mmHg in SBP or 10 mmHg in DBP across the BP range from 115/75 to 185/115 mmHg^14^. In patients with type 2 diabetes and hypertension, a treatment approach that included control of BP and glycemia significantly reduced the risk of cardiovascular complications and mortality.

SGLT transport links one glucose with one sodium ion for transportation into the proximal tubule cell. With inhibition of the SGLT2 protein, sodium reabsorption is reduced in the nephron, producing a mild diuretic effect^15,16^. Increased glucose in the filtrate will maintain an increased urine volume through osmotic diuresis. The possible mechanism of the decrease in blood pressure is that plasma volume reduction caused by both osmotic diuresis and natriuresis as well as body weight loss due to SGLT2 blocking^17^. Hypertension is an age-related disease. Previous studies have shown that arterial stiffness is the predominant cause of hypertension in the elderly^18,19^. Wu et al.^20^ have found that the age-related increase in blood pressure seemed to be largely mediated by age-related change in arterial stiffness status. Our study have shown that arterial stiffness have been improved as assessed by pulse wave velocity after empagliflozin therapy. This may be the major cause of blood pressure reduction in patients of empagliflozin group. Similarly, more and more studies have found that a mediterranean diet^21^, exercise training^22^, physical therapy^23^, or herb extract^24,25^ could reduce blood pressure by improving endothelial function. More importantly, Kawasoe et al.^17^ have addressed that the long-term BP-lowering effect related to SGLT-2 inhibitors was associated with the improvement of endothelial function. Moreover, our study has supported the above opinion that the improvement of endothelial function measured by flow-mediated dilation contributed to the blood pressure reduction related to empagliflozin.

Georgianos et al.^26^ analysed seven randomized controlled trials (RCTs) reporting treatment effects of SGLT-2 inhibitors on ambulatory BP. Compared with these seven trials, our study has not investigated the different effects between the high-dose and low-dose empagliflozin groups. Furthermore, we also have not taken hydrochlorothiazide as active comparator. These were the insufficiency of our study. However, the merit of our study was that we presented two of possible mechanisms in BP-lowering effect of empagliflozin which were the improvements of arterial stiffness and endothelial function making a case for the further studies.

## Conclusion

Empagliflozin was associated with significant and clinically meaningful reduction in BP versus placebo in Chinese elderly patients with type 2 diabetes and hypertension. The underlying mechanisms possibly at least in part were improvements of endothelial function and arterial stiffness associated with empagliflozin.
